# National Variation in the Use of Multiple Arterial Grafting in Isolated Coronary Artery Bypass Grafting in the United Kingdom

**DOI:** 10.1093/ejcts/ezaf402

**Published:** 2025-11-12

**Authors:** Jeremy Chan, Maria Comanici, Tim Dong, Pradeep Narayan, Daniel P Fudulu, Gianni D Angelini

**Affiliations:** Bristol Heart Institute, University of Bristol, Bristol BS2 8ED, United Kingdom; Bristol Heart Institute, University of Bristol, Bristol BS2 8ED, United Kingdom; Bristol Heart Institute, University of Bristol, Bristol BS2 8ED, United Kingdom; Bristol Heart Institute, University of Bristol, Bristol BS2 8ED, United Kingdom; Bristol Heart Institute, University of Bristol, Bristol BS2 8ED, United Kingdom; Bristol Heart Institute, University of Bristol, Bristol BS2 8ED, United Kingdom

**Keywords:** Multiple arterial grafting, Coronary artery bypass grafting, National variation

## Abstract

**Objectives:**

The last 2 decades have seen a reduction in the use of multiple arterial grafting (MAG) worldwide. The increase in risk profile in patients undergoing coronary artery bypass grafting (CABG) has been suggested as a cause for this limited use. This study aims to evaluate national variation at the surgeon and hospital level in the use of MAG while considering patients’ risk profiles.

**Methods:**

All patients who underwent first-time, elective/urgent, isolated CABG in the United Kingdom from 2010 to 2019 were included from the national adult cardiac surgery database. MAG was defined as the use of 2 or more arterial grafts. A 3-level multilevel logistic regression models (level 1: patients, level 2: surgeons, and level 3: hospitals) were used to estimate the variation in the use of MAG at each level.

**Results:**

Forty hospitals were identified, in which the MAG use ranged from 0% to 43.79%. A total of 135 978 patients were included in the study, of which 15 310 (11.3%) received MAG. Younger (odds ratio [OR]: 1.06, 95% confidence interval [CI]: 1.06-1.06, *P* < .001), male (OR: 1.14, 95% CI: 1.09-1.19, *P* < .001), and patients with fewer comorbidities and a higher socioeconomic status were more likely to receive MAG. After propensity score matching, there was no differences between patients who received single or MAG in in-hospital survival (0.8% vs 1.1%, *P* = .11), return to theatre for bleeding (3.3% vs 3.6%, *P* = .23), post-operative stroke (0.5% vs 0.3%, *P* = .08), and deep sternal wound infection (0.8% vs 0.8%, *P* = .66). Overall, surgeons’ and hospitals’ volumes were not associated with the use of MAG. However, surgeons with a higher volume of off-pump CABG were more likely to offer MAG (OR: 1.37, 95% CI: 1.31-1.42, *P* < .001). The interclass correlation coefficient was 0.31 at the surgeon level and 0.20 at the hospital level, implying 31% of the variability in the use of MAG is due to systematic differences between surgeons, and 20% due to systematic differences between hospitals.

**Conclusions:**

Our results demonstrate a considerable variation in both individual surgeons and hospital levels in the use of MAG. Young males with few comorbidities and higher socioeconomic status were more likely to be recipients of MAG. The use of multiple arterial grafts did not seem to increase the incidence of early in-hospital major complications.

## Graphical abstract

**Figure:**
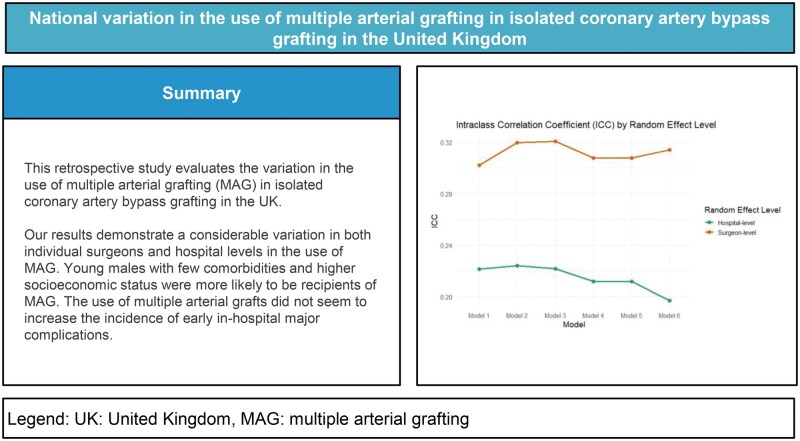

## INTRODUCTION

Over the past 2 decades, the global use of multiple arterial grafting (MAG) has significantly decreased.^1–3^ Despite growing evidence and guideline recommendations endorsing the benefits of MAG,^3–5^ their adoption remains restricted to specific surgeon experience and institutional volume.^6^^,^^7^

Concurrently, the patient population undergoing coronary artery bypass grafting (CABG) has seen notable changes, with significant increases in comorbidities and risk profiles (EuroScore II).^1^ Therefore, it could be argued that variation in MAG adoption is due to differences in patients’ comorbidities and risk profiles across the nation.

To account for these factors when assessing variation in MAG use, we employed a multilevel modelling (MLM) approach. MLM, also known as hierarchical linear modelling, can facilitate the analysis of patient-, surgeon-, and hospital-level factors to evaluate their impacts when applied to specific procedures. MLM helps to separate the variation due to individual patient factors from that due to surgeons or hospitals, providing a clearer and fairer understanding of where true differences in MAG use arise. We aimed to utilize MLM on a national dataset to investigate the variation in MAG use across the United Kingdom.

## METHODS

All patients who underwent elective or urgent isolated CABG from 2010 to April 2019 were extracted from the National Adult Cardiac Surgery Audit (NACSA) database. The NACSA database prospectively collects data on all primary heart operations carried out on National Health Service patients in the United Kingdom since April 1996. The definitions of database variables used and a description of the database were previously described.^8^ MAG was defined as the use of 2 or more arterial conduits.

The Index of Multiple Deprivation (IMD) decile values were recoded into quintiles, with quintile 1 indicating the most deprived and quintile 5 the least deprived. Missing IMD (<5%) values were imputed using the median value. Individual surgeons’ and hospitals’ volumes were categorized into 5 quartiles. Patients who underwent emergency or salvage CABG, previous cardiac surgery, received a single bypass graft, non-isolated CABG, and had missing data in the number and types of grafts, surgeons’ anonymized ID and hospital code (<2%) were excluded from the study.

Data are then input into the 6-model used in the MLM (see statistical analysis) to evaluate the variation of the use of MAG at patient-, surgeon-, and hospital-level. Intraclass correlation coefficient (ICC) was used to measure how much of the total variation in use of MAG is explained by clustering at a given level.

### Ethical statement

The study is part of a research project approved by the Health Research Authority (HRA) and Health and Care Research Wales (HCRW). As the study included retrospective interrogation of the NACSA database, the need for individual patient consent was waived off (HCRW) (IRAS ID: 278 171) in accordance with the research guidance. The study was performed in accordance with the ethical standards as laid down in the 1964 Declaration of Helsinki and its later amendments. The General Data Protection Regulations were strictly followed for the use of all data.

### Statistical analysis

Continuous variables were reported as median and interquartile range (IQR). Categorical variables were reported as frequencies and percentages. Pearson’s chi-squared test, Wilcoxon rank-sum test, and 1-way/multi-factor analysis of variance were used to compare 2 categorical variables, for comparison between means of 2 continuous, independent samples, and to compare between 3 continuous variables, respectively.

**Figure 1.**
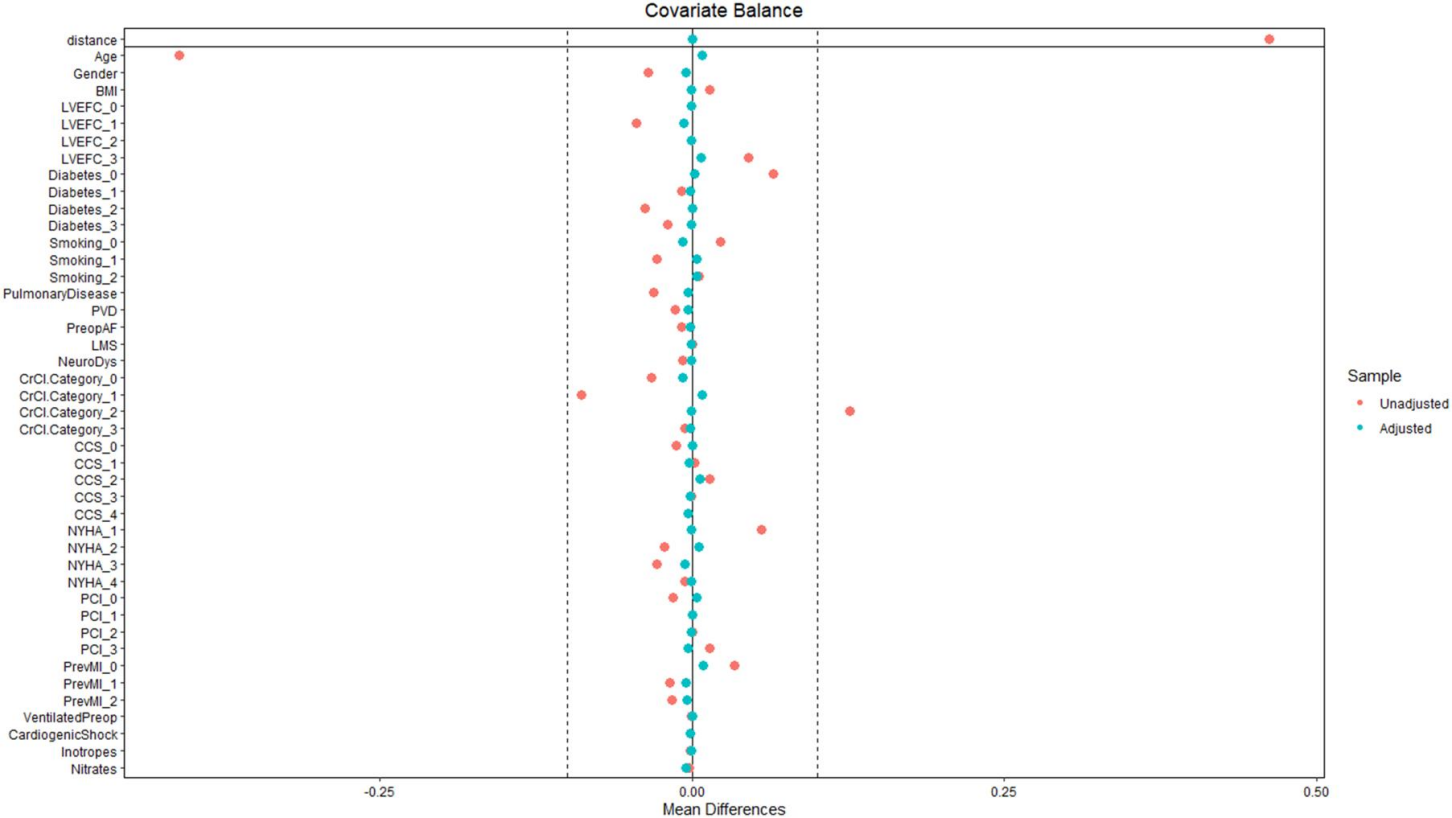
The Love Plot Visualizing the Effectiveness Before and After Propensity Score Matching. Abbreviations: AF: atrial fibrillation; BMI: body mass index; CCS: Canadian Cardiovascular Society; LMS: left main stem disease; LVEFc: left ventricular ejection fraction category; MI: myocardial infraction; NYHA: New York Heart Association; PCI: percutaneous coronary intervention; PVD: Peripheral vascular disease.

Propensity score matching (PSM) was performed to create a quasi-experimental design by balancing measured confounding factors between the 2 groups—single arterial grafting (SAG) and MAG. A 1:1 nearest neighbour matching without replacement with a calliper width of 0.2 standard deviation of the logit of the propensity scores was performed using the preoperative characteristics listed in **Table 1**. No formal adjustment for multiple comparisons was applied. Missing continuous variables data were imputed with the median value in the data after the application of exclusion criteria listed above. After matching, all standardized mean differences for the covariates were checked. An adequate balance was set to be below 0.1. The effectiveness of the PSM was visualized with a love plot showing the propensity score before and after the matching. This is shown in **Figure 1**.

**Table 1.** The Pre-operative Characteristics of Single and Multiple Arterial Groups Before and After Propensity Score Matching (PSM)

Pre-operative characteristics							
	SAG (*n* = 120 668)	MAG (*n* = 15 310)	*P* value	PSM—SAG (*n* = 15 292)	PSM—MAG (*n* = 15 292)	SMD	*P* value
Age (Year)	68 (60.8, 74.4)	63 (55.2, 70.7)	<.001	63 (55.2, 70.6)	63.1 (55.3, 70.7)	0.0079	.54
Gender (Male)	99 249 (82%)	13 128 (86%)	<.001	13 046 (85%)	13 114 (86%)	0.0127	.27
BMI	28.5(25.7, 31.4)	28.4 (25.7, 31.3)	.22	28.7 (25.8, 31)	28.4 (25.7, 31.3)	−0.0007	.24
CCS grade			<.001				.79
0	12 032 (10%)	1339 (8.7%)		1336 (8.7%)	1338 (8.7%)	0.0005	
1	10 779 (8.9%)	1400 (9.1%)		1425 (9.3%)	1397 (9.1%)	−0.0064	
2	46 001 (38%)	6057 (40%)		5952 (39%)	6050 (40%)	0.0131	
3	36 343 (30%)	4598 (30%)		4622 (30%)	4595 (30%)	−0.0039	
4	15 513 (13%)	1916 (13%)		1957 (13%)	1912 (13%)	−0.0089	
NYHA status			<.001				.57
1	33 163 (27%)	5065 (33%)		5052 (33%)	5051 (33%)	−0.0001	
2	60 099 (50%)	7283 (48%)		7191 (47%)	7281 (48%)	0.0118	
3	24 352 (20%)	2665 (17%)		2752 (18%)	2664 (17%)	−0.0152	
4	3054 (2.5%)	297 (1.9%)		297 (1.9%)	296 (1.9%)	−0.0005	
PreopAF	4155 (3.4%)	395 (2.6%)	<.001	417 (2.7%)	395 (2.6%)	−0.0273	.43
Previous MI							.15
No	58 687 (49%)	7968 (52%)		7821 (51%)	7959 (52%)	0.5114	
1	52 834 (44%)	6427 (42%)		6491 (42%)	6418 (42%)	−0.4245	
2 or more	9147 (7.6%)	915 (6%)		980 (6.4%)	915 (6%)	−0.0641	
Previous PCI			<.001				.85
0	102 823 (85%)	12 819 (84%)		12 755 (83%)	12 807 (84%)	0.8341	
1	385 (0.3%)	54 (0.4%)		53 (0.3%)	54 (0.4%)	0.0035	
2	1637 (1.4%)	211 (1.4%)		220 (1.4%)	209 (1.4%)	−0.0144	
3 or more	15 823 (13%)	2226 (15%)		2264 (15%)	2222 (15%)	−0.1481	
LVEF			<.001				.52
Good (>50%)	82 844 (69%)	11 502 (75%)		11 567 (76%)	11 678 (76%)	0.0006	
Moderate (31%-50%)	22 414 (19%)	2270 (15%)		3158 (21%)	2060 (13%)	−0.0365	
Poor (21%-30%)	10 110 (8.4%)	988 (6.5%)		558 (3.6%)	546 (3.6%)	−0.2065	
Very poor (≤20%)	5300 (4.4%)	550 (3.6%)		9 (<0.001%)	8 (<0.001%)	−0.7564	
Diabetes			<.001				.89
No	82 844 (69%)	11 502 (75%)		11 453 (75%)	11 484 (75%)	0.749	
Diet control	5300 (4.4%)	550 (3.6%)		575 (3.8%)	550 (3.6%)	−0.0376	
Drug control	22 414 (19%)	2270 (15%)		2268 (15%)	2270 (15%)	0.1483	
Insulin	10 110 (8.4%)	988 (6.5%)		996 (6.5%)	988 (6.5%)	−0.0651	
Smoking			<.001				.38
Non smoker	42 251 (35%)	5709 (37%)		5806 (38%)	5697 (37%)	−0.3797	
Ex-smoker	63 072 (52%)	7569 (49%)		7509 (49%)	7564 (49%)	0.491	
Current smoker	15 345 (13%)	2032 (13%)		1977 (13%)	2031 (13%)	0.1293	
Pulmonary disease	14 458 (12%)	1366 (8.9%)		1416 (9.3%)	1365 (8.9%)	−0.0926	.31
NeuroDys	3070 (2.5%)	278 (1.8%)	<.001	279 (1.8%)	278 (1.8%)	−0.0182	.97
Peripheral vascular disease	14 795 (12%)	1674 (11%)	<.001	1713 (11%)	1672 (11%)	−0.112	.45
Renal function			<.001				.2
Normal	69 789 (58%)	10 792 (70%)		10 776 (70%)	10 774 (70%)	−0.0704	
Mildly impaired	41 774 (35%)	3949 (26%)		3823 (25%)	3949 (26%)	0.25	
Moderately impaired	8104 (6.7%)	533 (3.5%)		641 (4.2%)	533 (3.5%)	−0.0419	
Dialysis preoperatively	1001 (0.8%)	36 (0.2%)		52 (0.3%)	36 (0.2%)	−0.0034	

Abbreviations: AF atrial fibrillation; BMI: body mass index; CI: confidence interval; ICC: intraclass correlation coefficient; IMD: Index of Multiple Deprivation; LVEF: left ventricular ejection fraction; LVEFc: left ventricular ejection fraction category; MI: myocardial infraction; OR: odds ratio; PVD: Peripheral vascular disease; SMD: Standardized Mean Difference.

A 3-level multilevel logistic regression model was fitted with patients at level 1, nested within surgeons (level 2), who were in turn nested within hospitals (level 3). The model included fixed effects for relevant patient-level covariates, including age, sex, diabetes, and other clinical characteristics. Random intercepts were specified for surgeons and hospitals to quantify variation in MAG use attributable to surgeons’ and institutions’ levels, respectively. The use of MLM in cardiac surgery using the national registry and its interpretation was previously discussed by Sanagou et al.^9^

To examine the factors influencing MAG use while accounting for clustering, a stepwise modelling approach was employed. Model 1 (null model) included only random intercepts for hospitals and surgeons to establish baseline variation in MAG use. Model 2 adjusted for baseline clinical risk using patients’ risk profile variables. Model 3 incorporated socioeconomic status via IMD quintiles. Model 4 further adjusted for procedural strategy (on-pump vs off-pump CABG). Model 5 included surgeon volume (quartiles of individual surgeon case volume over the study period). Model 6, the final model, added hospital volume (quartiles of institutional CABG volume). ICC was used to assess the degree of between-patients, surgeon and hospital variation in the use of MAG.

Model comparison was conducted using likelihood ratio tests to evaluate the incremental contribution of covariates at each stage. Fixed effects were reported as odds ratios (ORs) with 95% confidence intervals (CIs), and variance components were used to calculate ICCs to quantify between-surgeon and between-hospital variation.

R (Version 4.2.3, R Foundation for Statistical Computing, Vienna, Austria) and R Studio (Version 1.4.1103, RStudio, PBC) were used to perform statistical analysis. Graphs and tables were created using R (Version 4.2.3, R Foundation for Statistical Computing, Vienna, Austria) and Microsoft Office 365 (Version 16.0.14026, Microsoft Corporation, Washington, USA).

## RESULTS

**Figure 2.**
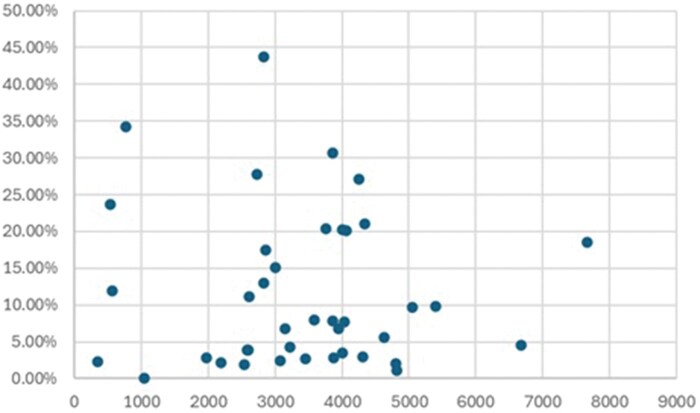
The Total Number of Isolated Coronary Artery Bypass Grafting Performed in Each Centre (x-Axis) and the Proportion of Multiple Arterial Grafting Performed (y-Axis). Each dot represents a single centre

Forty hospitals were identified, in which the MAG use ranged from 0% to 43.79% (**Figure 2**). The 3 highest volume centres performed 7662, 6680, and 5393 procedures and had a MAG rate of 18.6%, 4.54%, and 9.83%, respectively.

A total of 135 978 patients were included in the study, with the median age of 67.5 (first and third quartile: 60.1, 74.1) years and 83% were male. The median EuroScore II was 1.19 (first and third quartile: 0.82, 1.92). The use of MAG rates was 11.3% (*n* = 15 310) during the study period.

After PSM, there was no differences between patients who received single or MAG in in-hospital survival (0.8% vs 1.1%, *P* = .11), return to theatre for bleeding (3.3% vs 3.6%, *P* = .23), post-operative stroke (0.5% vs 0.3%, *P* = .08), and deep sternal wound infection prior to discharge from the index operation (0.8% vs 0.8%, *P* = .66). **Tables 1** and **2** shows the pre-operative, intraoperative, and post-operative characteristics before and after PSM.

**Table 2.** The Intra- and Post-operative Outcome Between SAG and MAG After Propensity Score Matching (PSM)

Characteristics	SAG (*n* = 120 668)	MAG (*n* = 15 310)	*P* value	PSM-SAG (*n* = 15 292)	PSM—MAG (*n* = 15 292)	*P* value
Use of CPB	105 414 (90%)	9275 (68%)	<.001	13 428 (91%)	9260 (67%)	<.001
CPB time (mins) (1st, 3rd Q)	83 (64, 103)	76 (49, 100)	<.001	83 (64, 103)	76 (49, 100)	<.001
XClamp time (mins) (1st, 3rd Q)	49 (36,63)	48 (30,65)	<.001	49 (36,63)	48 (30,65)	<.001
Number of grafts			<.001			<.001
2	27 912 (23%)	4051 (26%)		3588 (23%)	4043 (26%)	
3	62 117 (51%)	6874 (45%)		7657 (50%)	6869 (45%)	
4	27 000 (22%)	3486 (23%)		3522 (23%)	3481 (23%)	
5	3354 (2.8%)	769 (5%)		479 (3.1%)	769 (5%)	
6	285 (0.2%)	130 (0.8%)		46 (0.3%)	130 (0.9%)	
Mortality	1405 (1.2%)	146 (1%)	.03	122 (0.8%)	146 (1.0%)	.11
RTT	4405 (4%)	529 (3.6%)	.03	472 (3.3%)	529 (3.6%)	.23
Neurological events			<.001			.08
TIA	438 (0.4%)	35 (0.3%)		45 (0.3%)	35 (0.3%)	
CVA	600 (0.6%)	47 (0.3%)		66 (0.5%)	47 (0.3%)	
Dialysis	2038 (1.9%)	220 (1.5%)	.007	172 (1.2%)	220 (1.5%)	.03
DSWI (in hospital)	782 (1.1%)	76 (0.8%)	.05	73 (0.8%)	76 (0.8%)	.66

Abbreviations: AF: atrial fibrillation; BMI: body mass index; CCS: Canadian Cardiovascular Society; LMS: left main stem disease; LVEF: left ventricular ejection fraction; MAG: multiple arterial grafting; MI: myocardial infraction; NeuroDys: neurological dysfunction; NYHA: New York Heart Association; PCI: percutaneous coronary intervention; SAG: single arterial grafting.

### Multilevel modelling

Younger (OR: 1.06, 95% CI: 1.06-1.07, *P* < .001), male patients (OR: 1.12, 95% CI: 1.07-1.18, *P* < .001), and patients with fewer comorbidities and a higher socioeconomic status were more likely to receive MAG. Surgeons and hospitals with a higher volume of off-pump CABG were more likely to offer multiple arterial grafts (OR: 1.37, 95% CI: 1.31-1.42, *P* < .001), while individual surgeons’ volumes were not associated with the use of MAG.

The interclass correlation coefficient was 0.31 (surgeon level) and 0.20 (hospital level), implying 31% of the variability (in the use of MAG) is due to differences between surgeons (independent of the patients and hospital), and 20% due to systematic differences between hospitals (independent of the patients and surgeons) (**Table 3**). Patient-level factors or other unaccountable factors explain the remaining variation.

**Table 3.** The Multilevel Multivariable Logistic Regression of the Use of Multiple Arterial Grafting in Isolated Coronary Artery Bypass Grafting in the United Kingdom

	Model 1 (null)	Model 2	Model 3	Model 4	Model 5	Model 6
Characteristics	OR (95% CI)	OR (95% CI)	OR (95% CI)	OR (95% CI)	OR (95% CI)	OR (95% CI)
Level 1: Patient						
Age		0.94 (0.94-0.94)^***^	0.94 (0.94-0.94)^***^	0.94 (0.93-0.94)^***^	0.94 (0.93-0.94)^***^	0.94 (0.93-0.94)^***^
Gender						
Male		Reference	Reference	Reference	Reference	Reference
Female		0.86 (0.81-0.91)^***^	0.87 (0.82-0.92)^***^	0.88 (0.82-0.93)^***^	0.88 (0.82-0.93)^***^	0.88 (0.82-0.93)^***^
BMI		0.99 (0.99-1.0)^**^	0.99 (0.99-1.0)^**^	0.99 (0.99-1.0)^**^	0.99 (0.99-1.0)^**^	0.99 (0.99-1.0)^**^
LVEF		1.09 (1.06-1.12)^***^	1.08 (1.06-1.12)^***^	1.07 (1.04-1.10)^***^	1.07 (1.04-1.10)^***^	1.07 (1.04-1.10)^***^
Urgency						
Elective		Reference	Reference	Reference	Reference	Reference
Urgent		0.93 (0.89-0.97)^**^	0.93 (0.89-0.97)^**^	0.92 (0.88-0.97)^**^	0.92 (0.88-0.97)^**^	0.92 (0.88-0.97)^**^
Diabetes		0.83 (0.82-0.85)^***^	0.84 (0.82-0.86)^***^	0.83 (0.82-0.85)^***^	0.83 (0.82-0.85)^***^	0.83 (0.82-0.85)^***^
Smoking		0.86 (0.84-0.89)^***^	0.87 (0.85-0.90)^***^	0.88 (0.85-0.91)^***^	0.88 (0.85-0.91)^***^	0.88 (0.85-0.91)^***^
Pulmonary disease		0.82 (0.76-0.88)^***^	0.82 (0.77-0.89)^***^	0.80 (0.74-0.86)^***^	0.80 (0.74-0.86)^***^	0.80 (0.74-0.86)^***^
Peripheral vascular disease		1.03 (0.96-1.11)	1.04 (0.97-1.12)	1.05 (0.98-1.13)	1.05 (0.98-1.13)	1.05 (0.98-1.13)
Pre-operative AF		1.04 (0.91-1.18)	1.04 (0.91-1.18)	1.07 (0.93-1.22)	1.07 (0.93-1.22)	1.07 (0.93-1.22)
Neurological dysfunction		0.88 (0.76-1.02)	0.89 (0.76-1.04)	0.84 (0.71-0.98)^*^	0.84 (0.71-0.98)^*^	0.84 (0.71-0.98)^*^
Creatinine clearance						
Normal		Reference	Reference	Reference	Reference	Reference
Mild		1.18 (1.05-1.32)^**^	1.17 (1.05-1.31)^**^	1.18 (1.05-1.32)^**^	1.18 (1.05-1.33)^**^	1.18 (1.05-1.33)^**^
Moderate		1.35 (1.20-1.51)^***^	1.33 (1.19-1.50)^***^	1.34 (1.18-1.51)^***^	1.34 (1.18-1.51)^***^	1.34 (1.18-1.51)^***^
Dialysis		0.26 (0.17-0.38)^***^	0.26 (0.17-0.38)^***^	0.27 (0.18-0.41)^***^	0.27 (0.18-0.41)^***^	0.27 (0.18-0.41)^***^
Previous MI		0.9 (0.87-0.94)^***^	0.9 (0.87-0.94)^***^	0.9 (0.86-0.93)^***^	0.9 (0.86-0.93)^***^	0.9 (0.86-0.93)^***^
Indices of deprivation						
IMD 1			Reference	Reference	Reference	Reference
IMD 2			1.12 (1.04-1.21)^**^	1.13 (1.04-1.22)^**^	1.13 (1.04-1.22)^**^	1.13 (1.04-1.22)^**^
IMD 3			1.23 (1.14-1.32)^***^	1.25 (1.16-1.35)^***^	1.25 (1.16-1.35)^***^	1.25 (1.16-1.35)^***^
IMD 4			1.27 (1.18-1.37)^***^	1.27 (1.18-1.38)^***^	1.27 (1.18-1.38)^***^	1.27 (1.18-1.38)^***^
IMD 5			1.30 (1.20-1.40)^***^	1.30 (1.20-1.41)^***^	1.30 (1.20-1.41)^***^	1.30 (1.20-1.41)^***^
Level 2: Surgeon						
Use of cardiopulmonary bypass				0.63 (0.58-0.69)^***^	0.64 (0.58-0.69)^***^	0.63 (0.58-0.69)^***^
Surgeon volume						
First Q					Reference	Reference
Second Q					1.25 (0.94-1.67)	1.25 (0.95-1.65)
Third Q					1.10 (0.78-1.54)	1.13 (0.82-1.57)
Fourth Q					1.27 (0.86-1.87)	1.28 (0.88-1.87)
Level 3: Hospital						
Hospital volume						
First Q						Reference
Second Q						0.79 (0.30-2.04)
Third Q						1.32 (0.48-3.64)
Fourth Q						0.47 (0.15-1.42)
Intercept	0.04 (0.03-0.06)^***^	2.89 (1.79-4.69)^***^	2.57 (1.59-4.17)^***^	4.18 (2.58-6.76)^***^	3.76 (2.28-6.21)^***^	4.27 (2.20-8.30)^***^
Variance estimate	0.52	0.54	0.54	0.52	0.52	0.52
ICC						
Surgeon	0.3	0.32	0.32	0.31	0.31	0.31
Hospital	0.22	0.22	0.22	0.21	0.21	0.21

Abbreviations: CPB: cardiopulmonary bypass time; CVA: cerebrovascular accident; DSWI: deep sternal wound infection; MAG: multiple arterial grafting; Min: minutes; RTT: return to theatre; SAG: single arterial grafting; TIA: transient ischaemic attack; XClamp: cross clamp; *: <0.05; **: <0.01; ***: <0.001.

## DISCUSSION

Our findings indicate that, after accounting for patient characteristics, significant variation persists in the utilization of MAG at both the individual surgeon and hospital levels. Younger male patients with few comorbidities and higher socioeconomic status were more likely to receive MAG. Furthermore, the use of arterial grafts did not seem to increase the incidence of early in-hospital major complications.

Over the past 2 decades, despite growing evidence and guideline recommendations endorsing the benefits of MAG, its use has significantly decreased worldwide.^3–5^ MAG adoption remains restricted to specific surgeons and centres.^6^^,^^7^ A study by Velez and colleagues conducted a statewide survey to evaluate surgeons’ perception of the use of MAG. Half of the respondents reported non-routine MAG use in their practice, primarily due to the risk of potential postoperative complications. After linking the respondents’ data with the Society of Thoracic Surgeons patient data and considering the patients’ co-morbidities, there was a variation in the use of MAG at both the surgeons’ and hospitals’ levels. For example, 32% reported having a hospital MAG protocol, and this was associated with a higher MAG use. Such results were also observed in our study, where 50% of the variation was due to the surgeons’ and hospitals’ levels. Our results in the United Kingdom (11.3%) are somewhat similar to those in the United States, where 14% of patients received MAG in a study using the Society of Thoracic Surgeons Adult Cardiac Surgery Database between 2018 and 2019.^10^ In Canada, population-based data from Ontario (2008-2016) report 16.4% of patients received 2 arterial grafts and 6.1% received 3 arterial grafts.^6^ However, data from Australia and New Zealand showed a 54.7% use of MAG, suggesting variation is not only limited to surgeons and hospitals but potentially to the national and international level.^11^

There is no doubt that the surgeon and centre experience play a major role in the case selection and the outcome of MAG. A study by Schwann et al^12^ reported a higher operative mortality in patients receiving bilateral mammary artery than those receiving single mammary artery at low-experience centres in the United States. However, this was not observed in experienced centres, suggesting that experience matters in the use of MAG. A meta-analysis encompassing 27 000 patients from 34 studies reported an inverse relationship between centre volume in the use of MAG and long-term mortality, with lower-volume centres reporting a lower survival rate at both 5 and 10 years.^13^ In our analysis, after PSM, we did not find any significant difference in mortality and major early in-hospital complications associated with the use of MAG.

Patient risk profile undergoing CABG has been increasing for the past 2 decades in both the United Kingdom and worldwide.^1^ With an increasing risk profile, one could argue that this cohort of patients may not benefit from MAG. In a study analysing 26 000 patients using the New Jersey registry, the authors demonstrate that patients who received MAG had a better long-term survival and a lower incidence of repeat revascularization.^14^ However, the benefit of MAG on long-term survival was not observed in patients older than 70 years and those with poor left ventricular ejection fraction (≤30%) preoperatively.^14^ Gaudino et al^15^ examined the New York Cardiac Surgery Reporting System with approximately 64 000 patients and found similar findings. MAG was associated with a better long-term survival rate and reduction in repeat revascularization in low-risk patients only. Our study results also suggest that patients with fewer comorbidities were more likely to undergo MAG. These findings underscore the need to adjust for patient risk profiles using the MLM to account for variations in MAG utilization, thereby mitigating potential selection bias.

Enhanced recovery after surgery (ERAS) protocols in CABG are associated with improved postoperative outcomes such as reduced mechanical ventilation duration, shorter ICU and hospital stays, and lower rates of complications like bronchopneumonia, delirium, and acute kidney injury, without increasing mortality.^16^ These protocols are multimodal and focus on perioperative optimization, early mobilization, and minimizing surgical stress. The integration of ERAS protocols with MAG is not yet specifically addressed in the medical literature, but the available evidence suggests that ERAS could potentially be applied to patients undergoing MAG, as ERAS improves perioperative outcomes without increasing mortality or morbidity.^16^ Future studies integrating ERAS implementation status may clarify its impact on MAG utilization.

The technical complexity of MAG, including harvesting and anastomosis of arterial conduits such as the radial artery and bilateral internal thoracic arteries, requires advanced skill acquisition and experience. Insufficient exposure and lack of standardized training are major barriers to routine MAG use among trainees and practising surgeons.^17–19^ Venardos and colleagues conducted a survey with 84 thoracic surgery residents, with 76% and 35% claiming that they have no experience in radial artery and skeletonization mammary artery harvesting, respectively. Structured mentorship programs, where trainees work closely with experienced surgeons, facilitate the safe transfer of skills and help overcome the steep learning curve associated with MAG. Evidence demonstrates that training operations involving MAG can be performed safely by trainees under supervision, with outcomes comparable to those of experienced consultants, and that individual learning curves remain within acceptable error rates.^20^

In addition to surgical training, the implementation of hospital protocols or standardized pathways for MAG could increase the use of MAG. Adoption of hospital protocol for MAG was associated with increased utilization.^7^ This is likely due to explicit institutional support, standardized decision-making, and lower barriers to adoption MAG. Protocols could potentially facilitate multidisciplinary collaboration, streamline perioperative planning, and may include educational components or technical support, further increasing surgeon confidence and willingness to perform MAG.^7^

### Limitation

There are several limitations in our study. The NACSA database heavily relies on healthcare professionals’ input, and missing data are noted in some of the non-mandatory input. Overall, less than 2% of mandatory variables and <5% of IMD data were missing. However, for non-mandatory variables in post-operative outcomes, such as deep sternal wound infection, this could be higher (∼10%). The use of Y/T grafting and the specific details of individual graft configuration were also not recorded in the NACSA database. Hence, variation among different arterial strategies could not be analysed. However, the number and types of grafts are mandatory, and no significant discrepancies were observed. Despite the application of PSM, residual bias may still be present in the analysis, as the propensity-matched model can only account for measured confounders and not for unmeasured confounders (eg, frailty). MLM is powerful for handling nested data, but it has limitations. Large sample sizes are required at each level to minimize bias, which limits the use of this method to small datasets. Lastly, the absence of follow-up data in the NACSA database, which includes survival rates, the need for repeat revascularization, and the rate of major adverse cardiac events, is another limitation to evaluate the benefits of MAG fully. Therefore, our analysis focuses primarily on short-term outcomes and variation in practice patterns.

## CONCLUSION

Our results demonstrate a considerable variation at both the surgeons’ and hospitals’ levels in the use of MAG in isolated CABG in the United Kingdom. The use of MAG was more common in young males with few comorbidities and higher socioeconomic status. The use of MAG was not associated with an increase in the incidence of early in-hospital major complications. The substantial unwarranted variation suggests opportunities for quality improvement to standardize practice, including at both the surgeon and hospital levels.

## Data Availability

The data underlying this article were provided by the National Institute for Cardiovascular Outcomes Research by permission. Data will be shared on request to the corresponding author with permission from the National Institute for Cardiovascular Outcomes Research.
